# Data that matter: what outcomes matter to patients with congenital heart disease and their carers and which should be routinely measured? A qualitative asynchronous online discussion forum

**DOI:** 10.1136/bmjopen-2025-111466

**Published:** 2026-09-25

**Authors:** Jo Wray, Katherine L Brown, Fiona Kennedy, Christina Pagel, Sonya Crowe

**Affiliations:** 1 Heart and Lung Directorate, Great Ormond Street Hospital for Children NHS Foundation Trust, London, UK; 2 Barts Health NHS Trust, London, UK; 3 Clinical Operational Research Unit, University College London, London, UK

**Keywords:** Quality in health care, Patient Preference, QUALITATIVE RESEARCH

## Abstract

**Abstract:**

**Objectives:**

We wanted, as part of a wider study, to elicit perspectives of patients with congenital heart disease (CHD) and/or their parents/carers about which outcomes of CHD and/or cardiac surgery matter and what should be measured and reported.

**Design:**

Qualitative, closed, asynchronous, online discussion forums underpinned by an interpretivist framework.

**Setting and participants:**

Three patient charities in the UK set up and moderated separate online forums for adult patients with CHD/carers, adolescent patients with CHD and parents/carers of children and young people with CHD.

**Analysis:**

Thematic analysis using a codebook approach.

**Results:**

There were five forums in total; 343 participants signed up, two-thirds of whom were adult patients (n=235; 69%). Four themes reflecting different types of outcomes were identified: clinical outcomes related to surgery (eg, diagnosis-based survival, complications, readmission and reintervention rates); other clinical outcomes (eg, long-term survival, use of medications, pregnancy risks and outcomes); outcomes related to service provision (eg, availability of support services, cancellations, delayed follow-up); and patient-reported outcomes (eg, quality of life, mental health outcomes).

**Conclusions:**

Participants identified a range of outcomes about which they wanted more information but within the context of an individualised approach to how much, when and what information is provided. The importance of achieving a balance between knowing so much that it has a detrimental impact on mental health and not knowing enough so that patients/carers are unable to make informed decisions was emphasised.

**Strengths and limitations of this study**

- Data collection methods (asynchronous discussion forums on Facebook) facilitated accessibility for participants and resulted in a large sample of geographically and clinically diverse participants of different ages.
- Use of social media to collect data supports participation by those who might otherwise not engage in research.
- Most participants were female and ethnic diversity was limited.
- The study design precluded participation from those who were unable to read English, use social media or were unfamiliar with, or without access to, appropriate technology.

## Background

 In England, 4000–5000 babies are born with congenital heart disease (CHD) each year, with a relatively stable incidence of 0.8% of all live births.^1 2^ More than 95% now survive to adulthood,^3^ about half of whom will have undergone at least one cardiac procedure in childhood.^4^ CHD requires lifelong follow-up, ideally under the oversight of a specialist centre, with the majority of conditions never being ‘cured’ and frequently necessitating a complex regimen of medications, procedures and clinical appointments^5–7^; more than 20% of those who require surgery in childhood require further procedures in adulthood.^8^ Approximately 7000 CHD procedures (including surgery and cardiac catheters) in children and 4000 CHD procedures in adults are carried out each year in the UK (excluding Scotland).^9^ The number of procedures in adults will likely continue to increase as more patients with complex CHD live longer.^10^

Since 2000, early postoperative outcomes of paediatric cardiac surgery in England have been recorded and reported by the National Congenital Heart Diseases Audit.^9^ Collection of such data has provided valuable and reliable evidence of improving early survival rates and increasing proportions of children with more complex CHD and comorbidities being operated on at younger ages. While the quality of CHD services in England has traditionally been measured by 30-day survival following cardiac intervention in childhood,^11^ this is no longer considered an adequate proxy for quality in CHD services.^7 12–14^ A review of CHD services^14^ called for reporting of quality using a wider range of outcomes and metrics, a view supported by stakeholder groups including patients, families and clinicians, which would enable service quality to be judged more comprehensively as well as the impact (short and long-term) of CHD on patients to be more meaningfully evaluated. While some outcomes have been suggested based on research and service reviews, such as longer-term mortality,^14^ complications^15^ and reoperations,^16^ there are many challenges in operationalising these suggestions such as differences in how diagnoses and procedures are coded and variability between specialist centres in criteria for intervention. Consequently, there is a lack of agreement about which outcomes should be measured, how they should be defined and for which groups of patients. Furthermore, consulting with patients and parents/carers and incorporating their views into recommendations is widely acknowledged as a key element of service improvement, providing the unique user perspective.^17^

To address these gaps, we set up a national study (Congenital Heart Audit: Measuring Progress in Outcomes Nationally)^18^ to develop tools which could be used to routinely measure and monitor outcomes in CHD that are considered meaningful and important to stakeholders. One aspect, reported here, involved elicitation of the perspectives of patients with CHD and/or their parents/carers about which outcomes of CHD and/or cardiac surgery matter and what should be measured and reported (figure 1, objective 3).

**Figure 1:**
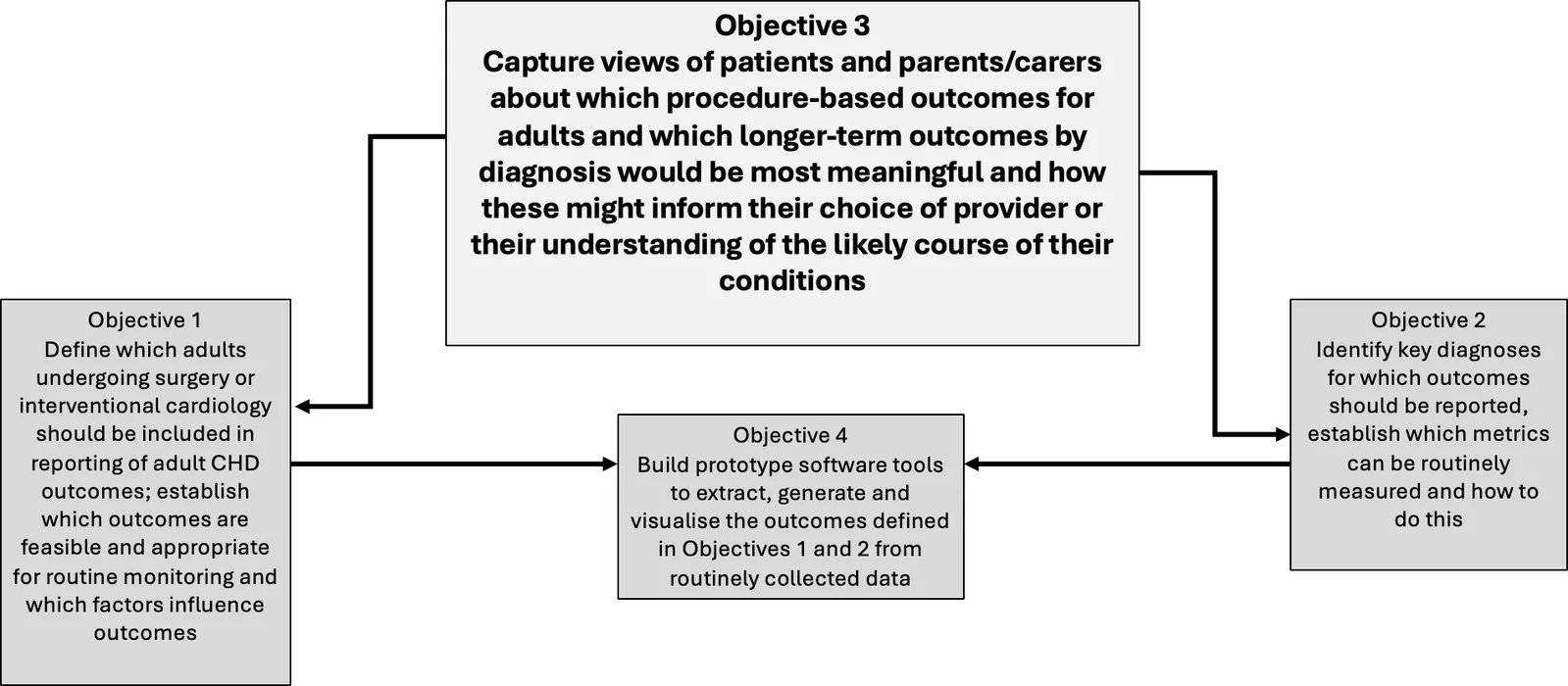
Phases of the wider CHAMPION study. CHAMPION, Congenital Heart Audit: Measuring Progress in Outcomes Nationally; CHD, congenital heart disease.

## Methods

### Objectives

To capture views of patients and parents/carers about which procedure-based outcomes for adults and which longer-term outcomes by diagnosis would be most meaningful and how these might inform their choice of provider or their understanding of the likely course of their conditions.

Detailed methods have been reported previously^19^ and are summarised in table 1.

**Table 1:** Summary of methods: participating charities, role of research team, forum set up, online safety, consent and data management and analysis

Charities	Three patient charities facilitated and moderated closed, asynchronous, online discussion forums via their Facebook pages: Little Hearts Matter Somerville Foundation Children’s Heart Federation Two charities ran one forum each representing parent/carers of children with CHD and adults with CHD; the third charity ran three forums, one for each of their participant groups (adolescents with CHD, adults with CHD and parents/carers)
Research team	Developed a series of questions focused on perceptions of experience and outcomes of care; questions revised based on feedback from Patient and Public Involvement (PPI) group (box 1).
Set up	Each charity advertised the discussion forums on their website Anyone interested in learning more was signposted to charity Facebook site to access further information about the overall study and what participation in the online discussion forum would involve Potential participants asked to provide some demographic information after which they were given access to the relevant discussion forum (based on whether they were a patient or parent/carer) Participants could join at any stage during the running of the forum and they had access to previously posted questions which they could also answer Forums ran for 12–24 weeks (during February–August 2021, dependent on responses and interruptions due to COVID-19).
Online safety	Each charity ran the discussion forums in line with a standard operating procedure coproduced with the research team which provided guidance about how to facilitate the forums and manage inappropriate posting or distressed users
Consent	As the discussion forums were anonymous and facilitated by the charities and no individual participant details were available to the research team, consent was not obtained from the participants. Consent was assumed from their engagement and participation. Participants were made aware that their anonymised quotes might be used in the research outputs. Charities were asked to put the following information on their website in relation to what would happen to the findings: “The results of the study will be published and shared with professionals at conferences and in journals. We will send a summary of the results to everyone who took part if they wish and we will also make a summary of the findings available on the CHF/LHM/SF website. The results of the study will be reported for the forum discussion as a whole and you will not be identified in any report or publication. However, information that you give us in relation to this project in any online posts may be anonymised and directly quoted in publications and presentations.”
Data management and analysis	Charities were responsible for removing any identifiable data before sending a single anonymised transcript of whole forum and aggregated demographic data to research team A code book approach was taken to analysis; three members of research team read and familiarised themselves with transcripts before meeting to discuss, create and agree themes All researchers were female and were experienced in the field of cardiac research; two (JW/KLB) had a clinical background in CHD Each researcher independently coded manuscript to agreed themes related to outcomes, additionally making a note of anything that did not fit with a theme In further meetings coding was discussed and any anomalies or conflicts in coding resolved by consensus Findings were discussed at study meetings, which included a patient co-researcher and charity representatives, to enhance trustworthiness and credibility of the analysis; final analysis was shared with PPI group for further assessment of credibility and trustworthiness

CHD, congenital heart disease; PPI, patient and public involvement.

Patient and/or parent/carer views were elicited via online closed, asynchronous discussion forums, an approach underpinned by an interpretivist framework and successfully used in earlier studies.^20 21^ Three UK patient charities hosted separate forums for adults, adolescents and parents/carers to capture diverse perspectives. Questions were developed by the research team (box 1) and refined by the patient and public involvement (PPI) group to ensure relevance and clarity. Participants were recruited via charity websites, completed demographic questionnaires and contributed to discussions over 12–24 weeks. Charities moderated forums using a standard operating procedure, including processes to identify and manage participant distress. Participation implied informed consent and participants were told that anonymised quotes could be used in publications. Following anonymisation, transcripts underwent codebook thematic analysis^22^ by three experienced female members of the research team, involving independent coding, codebook refinement, researcher consensus and PPI review. We used the Standards for Reporting Qualitative Research (SRQR) reporting guideline^23^ and checklist (online supplemental material) in the preparation of the manuscript.

Box 1 **Questions related to outcomes of importance that should be measured (adult patient forum)**

Questions for the parent/carer and teenager forums were similar, with minor wording changes to reflect those respondent groups (eg, designed to appeal to teenagers or wording appropriate for carers rather than patients). Charities could introduce questions to probe further.

1. The National Health Service (NHS) tries to monitor the quality of its services for congenital heart disease (CHD) and make this information transparent and available for everyone. For example, every year it publishes the proportion of children surviving at least 30 days after their heart surgery for each specialist hospital in the UK.
  - Did you know that this information is available for children’s surgery?
  - Do you think there should be something similar about survival after surgery for CHD in adulthood?
  - What about information on longer-term survival after surgery for CHD in childhood or adulthood? At the moment, results are published for 30-day survival following an operation but should longer-term outcomes be published? If so, what time period?
  - Are there any other things that you think they should measure and report publicly (Prompt: eg, complication rates, readmission to hospital, longer-term quality of life? In what ways would such information help you to make choices about your care or feel more confident in the services you receive?
2. Thinking now about what you want to know about your heart condition, is there information that you would like to know, but currently don’t, about:
  - What is likely to happen to you in the future in relation to your health?
  - How your condition might affect your lifestyle/work?
  - What health care services you are likely to need in the future?

At what stage would you want to know this information (eg, as soon as you transfer to adult services, before then, once you have been in adult services for a period of time)?

### Patient and public involvement

A CHD patient co-researcher led the PPI group for the overall project, which initially comprised three adults with CHD and one grandparent of a child with CHD. This group provided feedback on the content and language of questions for the online forums and also advised that individual forums should be run for adolescent and adult patients and parents/carers; revisions were made accordingly. Due to changes in individual circumstances, the PPI group altered towards the end of the project, with three new adults with CHD and one parent of a child with CHD joining the group. Following presentation of the overall project to this new group, with an emphasis on the online forum findings, a number of additional approaches to dissemination for patients, families and patient-facing organisations were suggested to increase accessibility of the findings to those with CHD. Suggestions included the development of a podcast/video interview with the research team discussing study results, an animation highlighting the main findings and an advocacy piece written with patients and families on outcomes that could be collected but currently are not.

## Results

Five forums were run across the three charities. Participant details are provided in table 2; the majority were White, female, adult patients with biventricular CHD.

**Table 2:** Characteristics of those who signed up to the online forums (n=343)

	Number (%)
Participants: adults with CHD	235 (69)
Young people with CHD	11 (3)
Parents/carers of adult patients with CHD	7 (2)
Parents/carers of children with CHD	90 (26)
Participant gender: male	33 (10)
Female	245 (71)
Unknown	65 (19)
Participant age group: <16 years	2 (1)
16–20	11 (3)
21–30	32 (9)
31–40	69 (20)
41–50	72 (21)
51–60	65 (19)
61+ years	15 (4)
Unknown	77 (22)
Age group of person with CHD: 0–1 years	9 (3)
2–5 years	17 (4)
6–10 years	15 (4)
11–15 years	8 (2)
16–18 years	9 (3)
19+ years	238 (69)
Unknown	47 (14)
Participant ethnicity: White	326 (95)
Non-White	5 (1)
Unknown	12 (3)
Location of specialist service: England (North East)	11 (3)
England (North West)	26 (6)
England (Yorkshire and the Humber)	20 (6)
England (East Midlands)	14 (4)
England (West Midlands)	47 (14)
England (East of England)	9 (3)
England (London)	75 (22)
England (South East)	28 (8)
England (South West)	27 (8)
Wales	8 (2)
Scotland	24 (7)
Northern Ireland/other	15 (4)
Unknown/none	39 (11)
Location of home: England (North East)	13 (4)
England (North West)	43 (13)
England (Yorkshire and the Humber)	22 (6)
England (East Midlands)	20 (6)
England (West Midlands)	36 (10)
England (East of England)	23 (7)
England (London)	22 (6)
England (South East)	57 (17)
England (South West)	46 (13)
Wales	21 (6)
Scotland	25 (7)
Northern Ireland/other	8 (2)
Unknown	7 (2)
Complexity of CHD: Single ventricle condition	121 (35)
Biventricular condition	216 (63)
Unknown	6 (2)

A number of participants chose not to provide some or any demographic information.

CHD, congenital heart disease.

Four themes reflecting different types of outcomes were identified: clinical outcomes related to cardiac surgery; other clinical outcomes; outcomes related to service provision; and patient-reported outcomes (figure 2). Themes are described below, with illustrative quotes.

**Figure 2:**
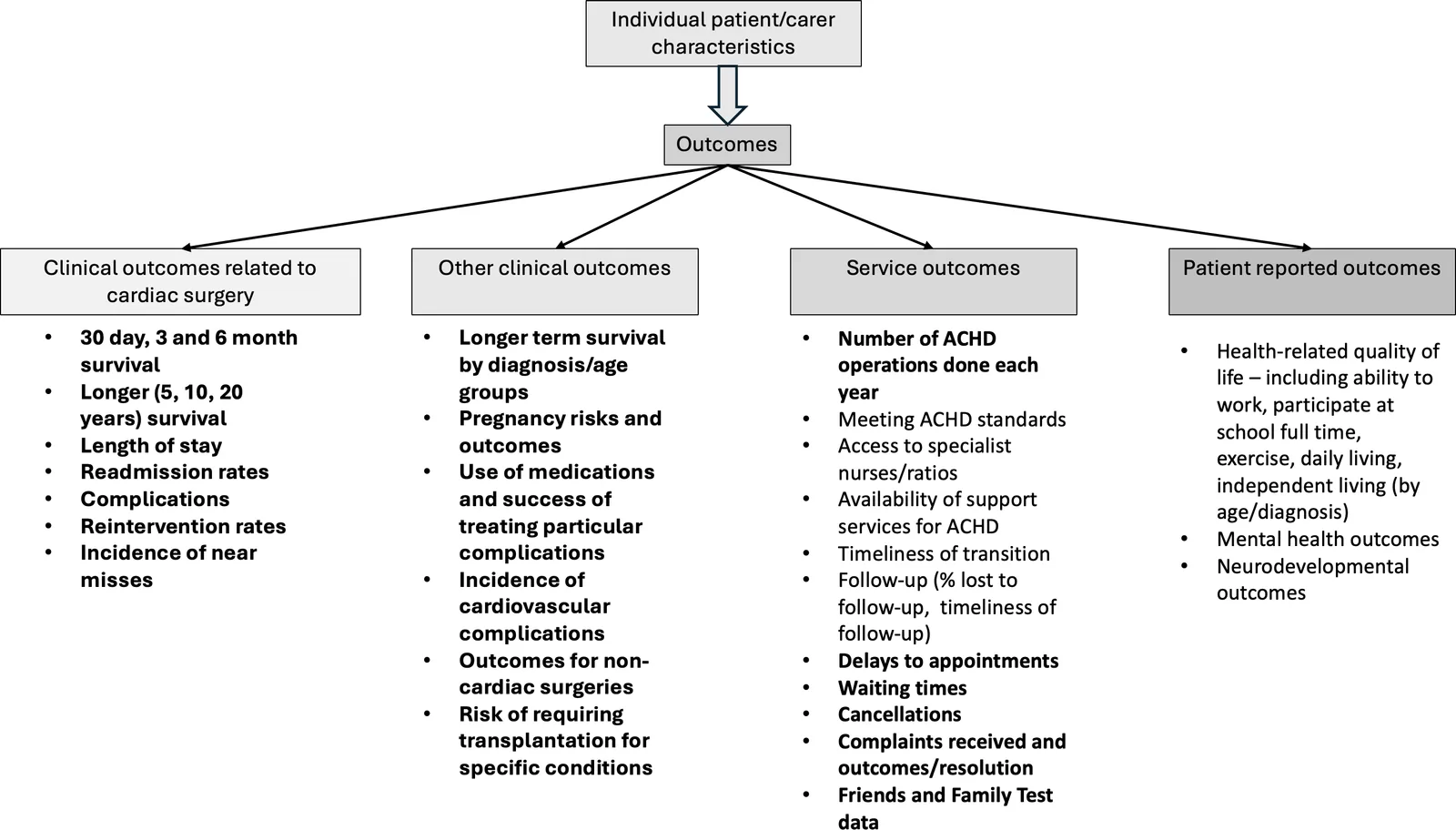
Themes related to outcomes that patients and carers want organisations to collect. ACHD, adult congenital heart disease.

### Clinical outcomes related to cardiac surgery

While most participating adults with CHD did not know about the data published on 30-day survival following cardiac surgery, parents of children with CHD reported looking for information prior to their child’s surgery and using the 30-day survival rates to inform their decision making. One parent commented, “I was acutely aware of the scrutiny of the paediatric cardiac surgical mortality statistics [in the media]. I felt instructed by the medical team as to which centre would operate but I needed to research this for myself…The medical team recommended a particular centre they routinely used. However I was aware that another centre had better results, I checked the data. I insisted on the other centre from the one recommended by the team. I felt the local team were perturbed by my approach but I had to know my son was in the best hands. It felt like the only thing I could do to improve his survival chances.” Parents commented that longer-term survival should be reported in addition to early survival rates, a view endorsed by adults with CHD: “How about 1yr, 5 yrs, 10 yrs, 25 yrs, and maybe even 50 yrs?” and “Re: survival from different procedures/surgery [in adulthood]—it would be nice to look at 1 month, 6 mths, 1 year, 5 and 10 years”. The need for help with interpretation of survival data, which may be complex, was also acknowledged, “…although I guess it might be difficult to show whether it is reintervention free” with another commenting that, “It is very hard for parents to access the meaning of this data”. Participants raised the importance of complex outcome events that may occur following a cardiac procedure, such as complications (eg, stroke) and reinterventions, particularly valve replacements and pacemakers. One adult described wanting information about complication rates and need for further surgery and how they might use this to inform their decision-making, “At least we could make an informed choice of who to trust our life with and whether having the surgery will improve/extend quality of life.” Participants articulated how information would “help people to know the risks involved and to put prospective treatments into perspective” and that also included adolescent patients, with one commenting, “They should have relevant statistics for procedure and that even [if] you are under 18 you should be told all this info”. Length of hospital stay, readmissions and near misses were also mentioned, sometimes in relation to individual hospitals as well as surgical interventions, “I believe that complication rates and readmissions would be good to know and to associate them to the certain hospitals where the procedures have happened throughout the UK…knowing these statistics could be helpful (inclusive of what hospitals the procedures were done at) as it would make for a better choice for patients, but mainly for the hospitals concerned who have the poorest statistics to have candour as to why there are failings. I would definitely need and want to have confidence before a major heart op and to understand my choices, pre-surgery.”

### Other clinical outcomes

Participants wanted information about cardiovascular complications related to the diagnosis itself such as arrhythmia and heart failure; the likelihood of heart transplantation occurring for those with specific conditions; and survival related to diagnosis by age group. Parents particularly commented that “it is important to have this information as soon as possible [after diagnosis].” Adults wanted to know about pregnancy risks and outcomes associated with different cardiac diagnoses, both from the perspective of the direct risk of being pregnant with a heart condition and the risks associated with the heritability of CHD, with the need for males as well as females to be involved in discussions about the latter. Non-cardiac surgery was also mentioned by a few participants, for example one commented, “It would be interesting to see how CHD patients fare when undergoing surgery elsewhere, such as gynae for example”. Complications related to diagnosis and occurrence of medication side effects were also mentioned, particularly by participants with a more complex diagnosis. For example, one parent described information she was given as her child approached the Fontan stage, “…liver complications, other surgery complications, meds and side effects…it would be good to have some kind of information when they’re starting nursery” while another parent commented about the timing of being given information and that it was overwhelming at the time and hard to remember years later, so a recap would be helpful.

### Service outcomes

Access to information related to services provided by specific hospitals was viewed as valuable for decision making. Adults discussed the importance of knowing the experience level of an individual surgeon and case volume of a treating centre, “I really want to know as an adult that if I require surgery or interventions that the people dealing with me have done this before. For example there might only be three operations doing something I might require per year in the whole of the UK, I want to know my centre has done those and it’s not 10 years since they did one!” Parents also commented on, “Having public information about the types of procedures and surgery that has taken place each year by each hospital, and the outcomes of these is probably the most useful information I would find helpful. That way I can easily double check if there is anyone with more experience, or undertaking something more frequently at a different hospital.” Participants wanted to know a hospital had “the expertise to deal with me” and the source of that information was clearly identified as being important, “Views from other patients are helpful but can be unreliable; reports about whole hospital performance may be helpful but may not offer a realistic picture of this particular service (this cuts both ways - might be a good service in a so-so hospital or a poor one in a top centre).” Another adult commented, “I want to know how many operations a particular surgeon or EP has done—how many ablations on someone with my CHD.” Others described generic service factors such as delays (eg, for diagnosis, treatment), availability of cardiac nurse support, waiting times, types of clinics, cancellations and complaints, “…whether they meet the ACHD standards and if not what they are doing to ensure they do. Also complaints figures (e.g. numbers made, resolved, patient satisfied with resolution*…*).“Friends & family” score (whether someone would recommend the service)…Publication of the information would give me confidence in the service and my decisions because I’d be able to see for myself what was happening, rather than just being told “oh yes, of course everyone is very good here”—which is generally true but occasionally not and greater transparency should pick these problems up sooner.”

Timeliness of transition to adult services, or loss to follow-up when this did not occur, and provision of information to enable patients to make informed choices about centres were described by both adults with CHD and parents. One young adult explained, “When I transitioned to adults it was very sudden. I always knew eventually I would have to transition. However at my last appointment in children’s it was just a matter of OK you are going to transition now. But I felt like adults welcomed very warmly. I was giving lots of info and booklets about adult cardiology unit as well.” In contrast, a parent stated,“There could be more information for the transitional ages…Adult units that are performing procedures and providing care for CHDers, and those outcomes…knowing all the who, where and what would reassure me. Specifically, I wouldn’t want her to get adult care from a cardiologist who has never seen an adult with a fontan, for example.”

### Patient-reported outcomes

Participants felt access to “quality of life info would help people” and providing such data at the time of diagnosis would “give hope”. They discussed the mental health implications of CHD, in terms of the impact of the condition and support, and parents specifically mentioned wanting data on long-term mental health outcomes. They also talked about children’s neurodevelopmental outcomes and their interest in accessing possible rates of these conditions in relation to a CHD diagnosis.

Across all described outcomes there was variation in quantity and type of information desired by different individuals, with some participants wanting facts about all outcomes, good and bad, to enable them to make decisions while for others there were clearly other implications of having information and needing to use that to make choices. As one parent stated, “And honestly in that moment when the diagnosis came, I’m not sure I would have known what to do with a choice”, while another parent commented, “I didn’t know this [30 day survival data] was available, and to be honest I’m not sure I’d read it even if I did. We have so much worry to deal with that seeing anything remotely negative just adds to that.” One parent suggested that a “layering effect of information would have helped at different stages” and adult patients also made reference to the timing of access to information, “Glad I wasn’t told everything when younger though. Would have spent years worrying about this last 9 hour complex op. I only had 18 months to worry instead. I think there should be a balance between knowing so much it impacts hugely on mental health and not knowing and unable to make informed decisions.”

## Discussion

People with CHD and their families want data about a range of outcomes related to their diagnosis, cardiac and non-cardiac treatment, services and psychosocial functioning which is tailored to their own individual information needs. Our findings resonate with those of the recent James Lind Alliance Priority Setting Partnership,^24^ which found that a number of priorities for research with both children and adults included holistic outcomes beyond early mortality, such as improving quality of CHD survivorship and reducing the impact of living with CHD. Currently, 30-day survival following surgery in childhood is the only fully risk-adjusted outcome metric routinely reported in the UK,^11^ although partially adjusted or non-adjusted outcomes are reported for complications and post-operative survival in adults. Longer-term survival and reintervention metrics for children from birth up to 10 years of age with one of nine key congenital heart defects have been developed,^25^ offering promise for the reporting of other clinical outcomes in the future. Furthermore, models for adult survival at 30 and 90 days and postsurgical complications have also been developed,^26^ informed by the forum data reported here. However, while some clinical cardiac outcome data that patients and carers want could, once defined, be gathered by specialist centres,^27 28^ other data are more challenging for hospitals/services to measure and collect or need to be obtained directly from patients. Barriers include logistical and governance issues, especially for the collection of identifiable data. Moreover, patients cannot be compelled to complete questionnaires about their quality of life, mental health or use of services, resulting in potentially incomplete data that are subject to response bias. A further challenge is getting agreement across CHD services about what should be measured, how and when. While implementation of electronic patient records and patient portals offers opportunities to streamline some of these processes, there are still many barriers to routine collection, storage and use of patient-reported outcome measures.^29^

In the era of the internet and social media, data are more accessible but with that has come ever greater expectations about what should be available. Some outcome data are unlikely ever to be collected, such as physician knowledge, but others would be feasible at a centre level, such as proportions of patients who are transitioned from paediatric to adult services and the timeliness of this.^30^ Outcome data are a key tool to empower patients and families in their decision-making at all stages of the care pathway but it is important to guard against the assumption that everyone wants to know everything about an increasing number of outcomes. Some patients and carers can feel overwhelmed by data and the pressure to use them to make critically important decisions. Unfettered access to data is therefore not always a good thing, particularly as accurate interpretation of complex outcome metrics can be challenging. Provision of information needs to be individualised not only in terms of the patient’s diagnosis and treatment pathway but also taking account of an individual’s desire for knowledge and level of detail that they want—how and how much information should an individual be given? A layered approach to information giving, the need for a ‘refresher’ and timing are all considerations; a web resource that people can explore at their own pace and which is layered in terms of information may offer part of the solution, as may key staff who are able to develop a relationship with patients and families over time and facilitate tailored information sharing to meet patients’/families’ needs.

### Strengths and limitations

This study allowed us to hear the views of a large number of people with CHD and their carers in a format that facilitated accessibility, resulting in a clinically and geographically diverse sample. However, the sample was not diverse with regard to ethnicity or gender, consistent with previous findings that females are more likely to use social media.^31^ Our approach excluded participants who could not read/write English or were not able or willing to engage with the necessary technology. CHD is more common in children of minoritised ethnicities living in more deprived areas^32^ and engaging such families in research is challenging.^24^ It is important that their views are heard and the research community needs to consider new ways of meaningfully engaging them. As we ran the discussion forums via three national charities it is likely that participants already in contact with the charities were more likely to engage, resulting in selection bias and limited generalisability of the findings. All participants were recruited via UK charities and were treated by the UK health system, further limiting generalisability outside the UK. We could not link demographic characteristics to responses and views of more vocal participants were likely to be over-represented although as we were interested in understanding the scope of outcomes that patients and carers thought were important and should be measured this was not a particular concern. Due to the anonymisation of transcripts it was not possible to know how many different participants responded to each question and how many questions each participant responded to. Of note, participants did endorse and comment/elaborate on others’ comments but probing of individual responses to elicit greater depth was not possible with the approach that we used. Finally, findings are potentially liable to interpretation/researcher bias, which we mitigated by three researchers undertaking analysis.

### Future directions

Providing relevant, timely and individualised outcome data is one element of delivering good care. However, to meet the information and decision-making needs of patients and carers, engagement of all stakeholders is required. We have suggested which outcomes can be measured and collected now, are feasible in the future or likely never going to be feasible, and whether the outcome data should be collected from centres or individual patients (figure 3). This work has informed the wider study objectives (figure 1). Future work is needed to reach consensus about how outcomes should be defined and measured, with standardisation across centres; we have previously demonstrated that this can be achieved for morbidities following paediatric cardiac surgery^33^ and in the current study we have taken the first step towards understanding what patients with CHD and carers want to know about outcomes.

**Figure 3:**
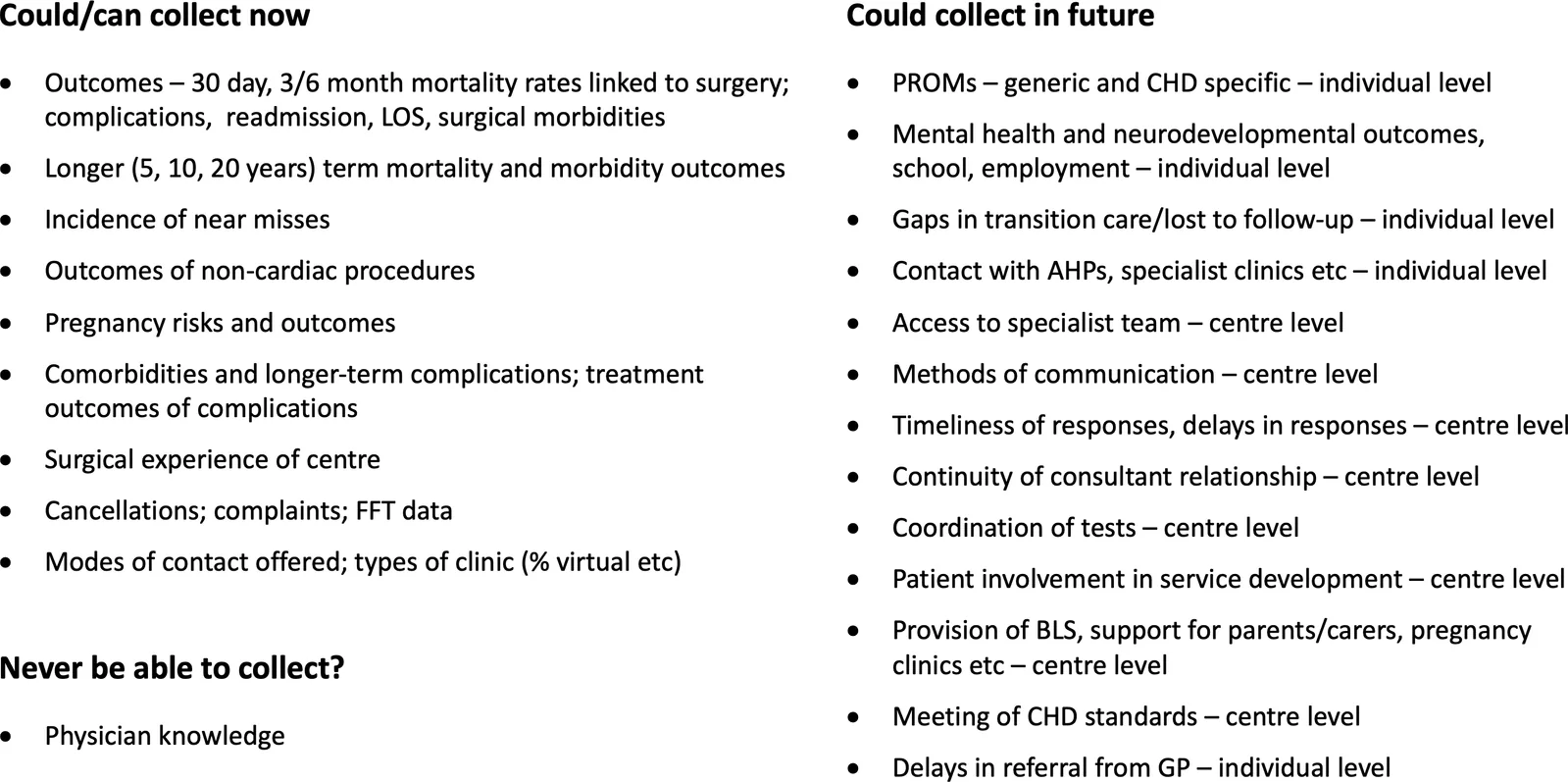
Outcomes that can be collected and/or reported now, in the future or probably never by centres and from individual patients. AHPs, allied health professionals; BLS, basic life support; CHD, congenital heart disease; FFT, Friends and Family Test; GP, general practitioner; LOS, length of stay; PROMs, patient-reported outcome measures.

## Supplementary material

10.1136/bmjopen-2025-111466 online supplemental file 1

## Data Availability

All data relevant to the study are included in the article or uploaded as supplementary information.

## References

[1] Dolk H, Loane M, Garne E (2011). Congenital heart defects in Europe: prevalence and perinatal mortality, 2000 to 2005. Circulation.

[2] NHS England (2017). Congenital heart disease services for adults and children: future commissioning arrangements. https://www.england.nhs.uk/wp-content/uploads/2017/11/06-pb-30-11-2017-congenital-heart-disease-services.pdf.

[3] Mandalenakis Z, Giang KW, Eriksson P (2020). Survival in Children With Congenital Heart Disease: Have We Reached a Peak at 97%?. J Am Heart Assoc.

[4] Cunningham D, Franklin R, Bridgewater B (2025). NICOR investigation of mortality from paediatric cardiac surgery in England 2009-12 2013. https://nicor4.nicor.org.uk/CHD/an_paeds.nsf/vwContent/.

[5] Razzaghi H, Oster M, Reefhuis J (2015). Long-term outcomes in children with congenital heart disease: National Health Interview Survey. J Pediatr.

[6] Coats L, Chaudhry B (2022). Ambulatory Care in Adult Congenital Heart Disease-Time for Change?. J Clin Med.

[7] Gurvitz M, Burns KM, Brindis R (2016). Emerging Research Directions in Adult Congenital Heart Disease: A Report From an NHLBI/ACHA Working Group. J Am Coll Cardiol.

[8] Verheugt CL, Uiterwaal CS, Vaartjes I (2017). Chance of surgery in adult congenital heart disease. Eur J Prev Cardiol.

[9] National Congenital Heart Disease Audit (NCHDA) (2025). 2025 annual report. https://www.nicor.org.uk/~documents/route%3A/download/3672.

[10] Brida M, Gatzoulis MA (2019). Adult congenital heart disease: Past, present and future. Acta Paediatr.

[11] Crowe S, Brown KL, Pagel C (2013). Development of a diagnosis- and procedure-based risk model for 30-day outcome after pediatric cardiac surgery. J Thorac Cardiovasc Surg.

[12] Eghtesady P, Brar AK, Hall M (2013). Prioritizing quality improvement in pediatric cardiac surgery. J Thorac Cardiovasc Surg.

[13] Gale CP, Weston C, Denaxas S (2012). Engaging with the clinical data transparency initiative: a view from the National Institute for Cardiovascular Outcomes Research (NICOR). Heart.

[14] NHS England (2015). New congenital heart disease review: final report. https://www.england.nhs.uk/wp-content/uploads/2015/07/Item-4-CHD-Report.pdf.

[15] Holst KA, Dearani JA, Burkhart HM (2011). Risk factors and early outcomes of multiple reoperations in adults with congenital heart disease. Ann Thorac Surg.

[16] Fiorentino F, Stickley J, Dorobantu D (2016). Early Reoperations in a 5-Year National Cohort of Pediatric Patients With Congenital Heart Disease. Ann Thorac Surg.

[17] Arya B, Glickstein JS, Levasseur SM (2013). Parents of Children with Congenital Heart Disease Prefer More Information than Cardiologists Provide. Congenit Heart Dis.

[18] Crowe S, Chester A, Ambler G (2019). Congenital heart audit: measuring progress in outcomes nationally (CHAMPION). https://fundingawards.nihr.ac.uk/award/PR-R20-0318-23001.

[19] Wray J, Pagel C, Coats L (2024). What does good care look like to people living with congenital heart disease in the 21st century? Qualitative online, asynchronous discussion forums. BMJ Open.

[20] Rajagopal V, Brown K, Pagel C (2020). Parental understanding of our communication of morbidity associated with paediatric cardiac surgery: a qualitative study. *BMJ Paediatr Open*.

[21] Wray J, Brown K, Tregay J (2018). Parents’ Experiences of Caring for Their Child at the Time of Discharge After Cardiac Surgery and During the Postdischarge Period: Qualitative Study Using an Online Forum. J Med Internet Res.

[22] Braun V, Clarke V (2021). Thematic analysis: a practical guide.

[23] O’Brien BC, Harris IB, Beckman TJ (2014). Standards for reporting qualitative research: a synthesis of recommendations. Acad Med.

[24] Drury NE, Herd CP, Biglino G (2022). Research priorities in children and adults with congenital heart disease: a James Lind Alliance Priority Setting Partnership. Open Heart.

[25] Brown KL, Huang Q, Espuny-Pujol F (2024). Evaluating Long-Term Outcomes of Children Undergoing Surgical Treatment for Congenital Heart Disease for National Audit in England and Wales. J Am Heart Assoc.

[26] Espuny-Pujol F, Pagel C, Ambler G (2026). New Models for Risk-Adjusted Monitoring of Postsurgical Complications and Mortality in Adult Congenital Heart Disease in England and Wales. Ann Thorac Surg.

[27] Dorobantu D-M, Espuny Pujol F, Kostolny M (2023). Arterial Switch for Transposition of the Great Arteries: Treatment Timing, Late Outcomes, and Risk Factors. *JACC Adv*.

[28] English KM, Espuny-Pujol F, Franklin RC (2025). Secundum atrial septal defect closure in adults in the UK. Eur Heart J Qual Care Clin Outcomes.

[29] Alarilla A, Sebire NJ, Keith J (2025). A scoping review of the electronic collection and capture of patient reported outcome measures for children and young people in the hospital setting. *PLOS Digit Health*.

[30] Espuny Pujol F, Franklin RC, Crowe S (2022). Transfer of congenital heart patients from paediatric to adult services in England. Heart.

[31] Ye L, Chen Y, Cai Y (2023). Gender Differences in the Nonspecific and Health-Specific Use of Social Media Before and During the COVID-19 Pandemic: Trend Analysis Using HINTS 2017-2020 Data. J Health Commun.

[32] Knowles RL, Ridout D, Crowe S (2017). Ethnic and socioeconomic variation in incidence of congenital heart defects. Arch Dis Child.

[33] Brown KL, Pagel C, Brimmell R (2017). Definition of important early morbidities related to paediatric cardiac surgery. Cardiol Young.

