# Supplementary material for: Data that matter: what outcomes matter to patients with congenital heart disease and their carers and which should be routinely measured? A qualitative asynchronous online discussion forum
Source: BMJ Open. 2026 Sep 25;16(9):e111466. doi: 10.1136/bmjopen-2025-111466 (PMC13630161; doi:10.1136/bmjopen-2025-111466)
Supplement: Supplementary file 1 [file bmjopen-2025-111466.reviewer_comments.pdf]

PEER REVIEW HISTORY

BMJ Open publishes all reviews undertaken for accepted manuscripts. Reviewers are asked to complete a checklist review form and are provided with free text boxes to elaborate on their assessment. These free text comments are reproduced below.

ARTICLE DETAILS

Title (Provisional)

Data that matter - what outcomes matter to patients with congenital heart disease and their carers and which should be routinely measured? A qualitative asynchronous online discussion forum

Authors

Wray, Jo; Brown, Katherine L; Kennedy, Fiona; Pagel, Christina; Crowe, Sonya

---

VERSION 1 - REVIEW

**Reviewer**	**1**
**Name**	**Menzies, Julie**
**Affiliation**	**Bristol Royal Hospital for Children, United Hospitals Bristol and Weston NHS Foundation Trust, Paediatric Intensive Care Unit**
**Date**	**16-Jan-2026**
**COI**	**None**

Thank you for the opportunity to review this paper on a really important topic. The paper is part of one workstream within a wider NIHR funded research project and they have already published a paper which had extensive review detailing the method and presenting results on a separate issue. The method is a really interesting way to engage with a wide range of people and hear a wide number of voices (although the authors do acknowledge the lack of diversity of those voices both in this paper and in their previously published work). It is great to see creative methods to find ways to hear seldom heard voices. I have only a few small comments to make below.

1. The authors acknowledge the change in PPIE participants over the life course of the study and the main additional contribution to the PPIE described in the previous paper, is about guidance on dissemination of results. It's unclear what the previous strategy was and how this is different now.

2. Results:

The authors have already published a paper reporting on other qualitative data gained during the same online forums so I have very little to add about the method or the results.

My only slight issue is whether there were some aspects which received a large volume of comments and other aspects which were only engaged with by a few people. For example I imagine most people would comment on clinical outcomes related to cardiac surgery, but how prevalent were concerns about other clinical outcomes? The limitations acknowledge that the views of more vocal participants were likely to be over represented, but it is hard at present to understand the level of engagement of people with the forums. Ie did most people comment on just one aspect? Did people tend to answer every question?

3. Another aspect that isn't captured (but I appreciate might not be possible from the anonymised way you received the transcripts) was about the interaction between participants in the forums. Did people show agreement or 'likes' with points individuals made? That might show wider consensus even if not many individuals comments.

4. Timeliness of transition to adult services, or loss to follow-up when this did not occur, and provision of information to enable patients to make informed choices about centres was described by both adults with CHD and parents, "There could be more information for the transitional ages…Adult units that are performing procedures and providing care for CHDers,and those outcomes…knowing all the who, where and what would reassure me. Specifically,I wouldn't want her to get adult care from a cardiologist who has never seen an adult with a fontan, for example."

The quote seems to tie with the last point about information provision. Does this also link to the timeliness of transition or loss to follow up? Unclear at present and this paragraph is currently a little heavy as it is all technically one sentence.

5. Discussion

I thought the discussion emphasised the importance of a tailored approach to information provision well. The concept of relationships and communication with clinicians was a core theme in the previous paper and seems to also link here, but there is really only reference to web resources. With all the discussion about individualisation and layered approaches, is there a role for key personnel involved with these families who might be well placed to facilitate this?

6. Further directions:

There is a figure summarising the work packages involved in CHAMPION, but the paper doesn't mention how this work is feeding into subsequent work within CHAMPION. The text says:

'Consensus is needed about how outcomes should be defined and measured, with standardisation across centres.' Is this further work being conducted within CHAMPION study?

**Reviewer**	**2**
**Name**	**Hueb, Whady**

**Affiliation**	**Universidade Federal de Sao Paulo**
**Date**	**16-Mar-2026**
**COI**	**None**

For the authors;

The study as presented has weaknesses and inconsistencies that should be considered.

Major problems:

1-TITLE: The title is inappropriate. It is colloquial. It should be changed.

2-ABSTRACT: The abstract does not conform to standard abstracts. It should be changed.

3-INTRODUCTION: The introduction should contain a literature review in accordance with the model of this study. It should contain everything that is known about the topic, everything that is not known, its similarities and controversies, and everything that one wants to know.

4-OBJECTIVES: The objectives should be clear and assertive.

5-METHODS: The methods should adhere to the rigor of the research. The following is observed:

A- The inclusion of participants occurred through Facebook groups administered by patient organizations, which generates several problems:

A1- participation restricted to digitally active individuals

A2- greater probability of inclusion of people more engaged with the disease

A3- possible over-representation of patients with higher educational or socioeconomic levels. Consequently, the results may not represent the general population of patients with CC.

B- Limited geographical representativeness: all participants are from the United Kingdom, which restricts generalization to other health systems.

Many identified outcomes, especially those related to service delivery, are heavily dependent on the British health system (NHS) model. In other contexts, such as mixed or private systems, priorities may be different.

C- Qualitative and Interpretivist Nature: The study is based on an interpretative approach, common in qualitative research, but this implies:

A1- absence of objective measurement

A2- interpretation dependent on the researchers

A3- possibility of interpretation bias in the thematic analysis

Furthermore, it is not clear in the abstract what qualitative analysis method was used (e.g., thematic analysis, grounded theory); whether there was independent double coding and finally, how thematic saturation was ensured.

These elements are fundamental to evaluating methodological robustness.

D- Unequal participation between groups: of the total participants; 69% were adult patients. This may lead to underrepresentation of pediatric perspectives, especially relevant in congenital heart disease, which is a disease frequently diagnosed in childhood.

E- Inherent limitation of the online forum format: The asynchronous forum format presents some methodological limitations:

A1- Impossibility of in-depth analysis of individual responses

A2- Less control over the quality of contributions

A3- Risk of influence among participants (conformity effect)

Furthermore, there is no guarantee that all participants have contributed in an equivalent manner.

RESULTS

The results reveal an important aspect: there is a discrepancy between the outcomes valued by researchers and those considered relevant by patients.

While traditional research prioritizes: mortality, complications, and reinterventions.

Patients and caregivers highlighted less relevant data: quality of life, psychological impact, uncertainty about the future, access to and organization of care, and adequate information for decision-making.

Another point highlighted is the balance between information and emotional impact. Many participants highlighted that: insufficient information hinders informed decisions; excessive information can generate anxiety and psychological suffering.

This aspect suggests the need for individualized medical communication strategies.

FINAL CONSIDERATIONS:

Clinical impact and applicability:

Direct applicability in clinical practice is limited because: it does not quantify the relative importance of each outcome; it does not establish measurable metrics; and it does not assess the association between outcomes and prognosis. Therefore, the results should be interpreted as exploratory and hypothesis-generating.

VERSION 1 - AUTHOR RESPONSE

Reviewer: 1
Dr. Julie Menzies, Bristol Royal Hospital for Children, United Hospitals Bristol and Weston NHS Foundation Trust
Comments to the Author:
Thank you for the opportunity to review this paper on a really important topic. The paper is part of one workstream within a wider NIHR funded research project and they have already published a paper which had extensive review detailing the method and presenting results on a separate issue. The method is a really interesting way to engage with a wide range of people and hear a wide number of voices (although the authors do acknowledge the lack of diversity of those voices both in this paper and in their previously published work). It is great to see creative methods to find ways to hear seldom heard voices. I have only a few small comments to make below.

RESPONSE: Thank you for your positive feedback.

1. The authors acknowledge the change in PPIE participants over the life course of the study and the main additional contribution to the PPIE described in the previous paper, is about guidance on dissemination of results. It's unclear what the previous strategy was and how this is different now.

RESPONSE: The original PPIE group provided guidance about the language and content of the questions for the online forum and the strategy also said that they would provide guidance about dissemination. Although the strategy did not change, the later members of the PPIE group focused on specific dissemination approaches and had suggestions related to accessibility of findings. We had not previously articulated approaches such as a podcast or animation and the group were particularly keen to be involved in writing an advocacy piece, which was not something we had considered previously. We have added text into the PPI section of the paper describing the approaches suggested by the group.

2. Results:
The authors have already published a paper reporting on other qualitative data gained during the same online forums so I have very little to add about the method or the results. My only slight issue is whether there were some aspects which received a large volume of comments and other aspects which were only engaged with by a few people. For example I imagine most people would comment on clinical outcomes related to cardiac surgery, but how prevalent were concerns about other clinical outcomes? The limitations acknowledge that the views of more vocal participants were likely to be over represented, but it is hard at present to understand the level of engagement of people with the forums. Ie did most people comment on just one aspect? Did people tend to answer every question?

RESPONSE: Thank you for this comment. The reviewer is correct is surmising that most people commented on clinical outcomes and that there were relatively few comments on non-cardiac outcomes in comparison, which we have clarified in the text. Unfortunately, due to the way the transcripts were submitted to us, we are unable to know the response pattern of individuals and how many participants responded to each question (some may have made more than one comment, sometimes in response to another participant's

comment) or how many questions each participant responded to. We have added more text to the limitations section to clarify this.

3. Another aspect that isn't captured (but I appreciate might not be possible from the anonymised way you received the transcripts) was about the interaction between participants in the forums. Did people show agreement or 'likes' with points individuals made? That might show wider consensus even if not many individuals comments.

RESPONSE: There was evidence of some participant interaction, with people endorsing others' comments or offering support if someone had described a distressing experience etc., but it is difficult due to the anonymisation to add anything particularly meaningful to the text about the interactions. However, we have now highlighted that some participants did endorse/comment on others' comments.

4. Timeliness of transition to adult services, or loss to follow-up when this did not occur, and provision of information to enable patients to make informed choices about centres was described by both adults with CHD and parents, "There could be more information for the transitional ages…Adult units that are performing procedures and providing care for CHDers,and those outcomes…knowing all the who, where and what would reassure me. Specifically,I wouldn't want her to get adult care from a cardiologist who has never seen an adult with a fontan, for example."
The quote seems to tie with the last point about information provision. Does this also link to the timeliness of transition or loss to follow up? Unclear at present and this paragraph is currently a little heavy as it is all technically one sentence.

RESPONSE: Thank you, we agree with the reviewer that this was an unclear paragraph and the two aspects of timeliness of transition and provision of information related to making choices about centres were somewhat intertwined. To clarify this, we have amended the paragraph to include an additional quote from a young adult about timeliness of transition.

5. Discussion
I thought the discussion emphasised the importance of a tailored approach to information provision well. The concept of relationships and communication with clinicians was a core theme in the previous paper and seems to also link here, but there is really only reference to web resources. With all the discussion about individualisation and layered approaches, is there a role for key personnel involved with these families who might be well placed to facilitate this?

RESPONSE: Thank you for the suggestion about a role for key staff involved with families – we agree that staff who have the opportunity to build a relationship with patients and families are well placed to share information tailored to the needs of the individual patient and family. We have added a further sentence to that effect.

6. Further directions:
There is a figure summarising the work packages involved in CHAMPION, but the paper doesn't mention how this work is feeding into subsequent work within CHAMPION. The text says:

'Consensus is needed about how outcomes should be defined and measured, with standardisation across centres.' Is this further work being conducted within CHAMPION study?

RESPONSE: Findings from the online forum have informed the wider study objectives, and we have clarified that in the text as well as making it clear that future work (beyond CHAMPION) needs to achieve consensus about how outcomes should be defined and measured.

Reviewer: 2
Dr. Whady Hueb, Universidade Federal de Sao Paulo
Comments to the Author:
For the authors;
The study as presented has weaknesses and inconsistencies that should be considered.
Major problems:
1-TITLE: The title is inappropriate. It is colloquial. It should be changed.
RESPONSE: We have added the study design to the title as requested by the Editor.

2-ABSTRACT: The abstract does not conform to standard abstracts. It should be changed.
RESPONSE: We have followed the abstract structure for *BMJ Open* but have rewritten the design and setting to include the study design more specifically, as requested by the Editor and added a section on the analysis.

3-INTRODUCTION: The introduction should contain a literature review in accordance with the model of this study. It should contain everything that is known about the topic, everything that is not known, its similarities and controversies, and everything that one wants to know.
RESPONSE: Thank you for these comments. We have included literature about congenital heart disease and the measurement of outcomes, highlighting the gap in the literature about what outcomes patients and their parents/carers think are important and should be measured, which is what we describe in this manuscript. We have contextualised the current manuscript in terms of the wider study of which this formed a part, outlined in Figure 1.

4-OBJECTIVES: The objectives should be clear and assertive.
RESPONSE: Thank you – we appreciate that these may not have been clear so have added a heading for objectives and added the specific objectives addressed in this manuscript.

5-METHODS: The methods should adhere to the rigor of the research. The following is observed:
A- The inclusion of participants occurred through Facebook groups administered by patient organizations, which generates several problems:
A1- participation restricted to digitally active individuals
A2- greater probability of inclusion of people more engaged with the disease
A3- possible over-representation of patients with higher educational or socioeconomic levels. Consequently, the results may not represent the general population of patients with CC.

B- Limited geographical representativeness: all participants are from the United Kingdom, which restricts generalization to other health systems.
Many identified outcomes, especially those related to service delivery, are heavily dependent on the British health system (NHS) model. In other contexts, such as mixed or private systems, priorities may be different.
C- Qualitative and Interpretivist Nature: The study is based on an interpretative approach, common in qualitative research, but this implies:
A1- absence of objective measurement
A2- interpretation dependent on the researchers
A3- possibility of interpretation bias in the thematic analysis
Furthermore, it is not clear in the abstract what qualitative analysis method was used (e.g., thematic analysis, grounded theory); whether there was independent double coding and finally, how thematic saturation was ensured.
These elements are fundamental to evaluating methodological robustness.
D- Unequal participation between groups: of the total participants; 69% were adult patients. This may lead to underrepresentation of pediatric perspectives, especially relevant in congenital heart disease, which is a disease frequently diagnosed in childhood.
E- Inherent limitation of the online forum format: The asynchronous forum format presents some methodological limitations:
A1- Impossibility of in-depth analysis of individual responses
A2- Less control over the quality of contributions
A3- Risk of influence among participants (conformity effect)
Furthermore, there is no guarantee that all participants have contributed in an equivalent manner.

RESPONSE: We recognise all of the points that the reviewer has made and have added further information to the limitations to further address some of these points. Whilst we acknowledge the limitations of the online forum format, we have successfully used this approach before to elicit the views of multiple participants including those who might otherwise not participate in research. As this is a qualitative approach we did not want to 'control' the quality of contributions or achieve equal participation between groups or individuals and we did not want to objectively measure anything. However, we have added the fact that findings are liable to interpretation bias. We have also added further information in the abstract about the approach to analysis. We also acknowledge the limited generalisability to participants in the UK and the UK health system – but would also highlight that our focus was the UK and the UK health system.

RESULTS
The results reveal an important aspect: there is a discrepancy between the outcomes valued by researchers and those considered relevant by patients.
While traditional research prioritizes: mortality, complications, and reinterventions.
Patients and caregivers highlighted less relevant data: quality of life, psychological impact, uncertainty about the future, access to and organization of care, and adequate information for decision-making.
Another point highlighted is the balance between information and emotional impact. Many participants highlighted that: insufficient information hinders informed decisions; excessive

information can generate anxiety and psychological suffering.
This aspect suggests the need for individualized medical communication strategies.

RESPONSE: Thank you for these comments. Whilst we agree that researcher and patient priorities are likely different, we do not agree that patients and carers highlighted less 'relevant' data – these are important and relevant to them and as such it is important that we measure these outcomes as well as more 'traditional' outcomes. We do agree, however, that individually tailored approaches to communication are needed.

FINAL CONSIDERATIONS:
Clinical impact and applicability:
Direct applicability in clinical practice is limited because: it does not quantify the relative importance of each outcome; it does not establish measurable metrics; and it does not assess the association between outcomes and prognosis. Therefore, the results should be interpreted as exploratory and hypothesis-generating.

RESPONSE: We agree that our findings do not quantify the relative importance of each outcome – this was not our intention, rather we set out to understand what outcomes are of importance to patients/carers and should be measured. As we have outlined, the next stage of this work is to reach consensus about how outcomes should be defined and measured, with the goal of achieving standardisation across centres.

We believe that the revisions have strengthened our manuscript and we hope that it will now be considered acceptable for publication in *BMJ Open.*

VERSION 2 - REVIEW

**Reviewer**	**1**
**Name**	**Menzies, Julie**
**Affiliation**	**Bristol Royal Hospital for Children, United Hospitals Bristol and Weston NHS Foundation Trust, Paediatric Intensive Care Unit**
**Date**	**08-May-2026**
**COI**	

Thank you for the opportunity to review this revised manuscript. The authors have addressed all of my comments / questions and I feel the revisions enhance the paper. I have no further comments / requests. I look forward to seeing this in print.

**Reviewer**	**2**
**Name**	**Hueb, Whady**
**Affiliation**	**Universidade Federal de Sao Paulo**

Date **27-Apr-2026**

COI

To the Authors

The study, as presented, displays an inappropriate format for a medical journal. The title is colloquial, there is no hypothesis, the objective is unclear, the results do not adhere to methodological rigor, and the discussion does not interpret the results. Additionally, there are important limitations, especially selection bias, limited representativeness, and the interpretative qualitative nature of the findings. This restricts the generalizability of the findings. Thus, the results should be considered exploratory and descriptive.

Major problems;

Unresolved issues:

1-TITLE: The title is inappropriate. It is colloquial. It should be changed.

RESPONSE: We have added the study design to the title as requested by the Editor.

Question: I think this response is inappropriate. Unless the editor is also a reviewer. Usually, the title should have clarity, precision, and scientific relevance.

As a reviewer, I consider the title to be like a newspaper headline.

2-ABSTRACT: The abstract does not conform to standard abstracts. It should be changed.

RESPONSE: We have followed the abstract structure for BMJ Open but have rewritten the design and setting to include the study design more specifically, as requested by the Editor and added a section on the analysis.

As a reviewer I consider unsatisfactory response. Again. I can't believe the editor agreed to change the abstract format.

3-INTRODUCTION: The introduction should contain a literature review in accordance with the model of this study. It should contain everything that is known about the topic, everything that is not known, its similarities and controversies, and everything that one wants to know.

RESPONSE: Thank you for these comments. We have included literature about congenital heart disease and the measurement of outcomes, highlighting the gap in the literature about what outcomes patients and their parents/carers think are important and should be measured, which is what we describe in this manuscript. We have contextualised the current manuscript in terms of the wider study of which this formed a part, outlined in Figure 1.

As a reviewer, I consider the question unanswered. The authors did not substantially change the introduction.

4-OBJECTIVES: The objectives should be clear and assertive.

RESPONSE: Thank you – we appreciate that these may not have been clear so have added a heading for objectives and added the specific objectives addressed in this manuscript.

The objectives are still unsatisfactory. They should be completely redone.

5-METHODS: The methods should adhere to the rigor of the research. The following is observed:

A- The inclusion of participants occurred through Facebook groups administered by patient organizations, which generates several problems............................

RESPONSE: We recognise all of the points that the reviewer has made and have added further information to the limitations to further address some of these points. Whilst we acknowledge the limitations of the online forum format, we have successfully used this approach before to elicit the views of multiple participants including those who might otherwise not participate in research. As this is a qualitative approach we did not want to 'control' the quality of contributions or achieve equal participation between groups or individuals and we did not want to objectively measure anything. However, we have added the fact that findings are liable to interpretation bias. We have also added further information in the abstract about the approach to analysis. We also acknowledge the limited generalisability to participants in the UK and the UK health system – but would also highlight that our focus was the UK and the UK health system.

Not answered: The authors must understand that the format of a medical publication has strict guidelines and does not allow for shortcuts. This study is not structured according to the methodological rigor namely: Hypothesis-Objective and Methods. Each of these has its own rules and does not accept changes according to the nature of the study. The fact that the material has already been published, in part, does not give a "free pass" for another publication.

6- RESULTS

The results reveal an important aspect: there is a discrepancy between the outcomes valued by researchers and those considered relevant by patients.

While traditional research prioritizes: mortality, complications, and reinterventions.

Patients and caregivers highlighted less relevant data: quality of life, psychological impact, uncertainty about the future, access to and organization of care, and adequate information for decision-making.

Another point highlighted is the balance between information and emotional impact. Many participants highlighted that: insufficient information hinders informed decisions; excessive information can generate anxiety and psychological suffering.

This aspect suggests the need for individualized medical communication strategies.

RESPONSE: Thank you for these comments. Whilst we agree that researcher and patient priorities are likely different, we do not agree that patients and carers highlighted less

‘relevant’ data – these are important and relevant to them and as such it is important that we measure these outcomes as well as more ‘traditional’ outcomes. We do agree, however, that individually tailored approaches to communication are needed.

This reviewer disagrees. Patients and caregivers are not medical professionals. They have different focuses. Therefore, the reader of a medical journal is a doctor. They want medical data. Information from caregivers or patients fails to provide technical information.

7-FINAL CONSIDERATIONS:

Clinical impact and applicability:

Direct applicability in clinical practice is limited because: it does not quantify the relative importance of each outcome; it does not establish measurable metrics; and it does not assess the association between outcomes and prognosis. Therefore, the results should be interpreted as exploratory and hypothesis-generating.

RESPONSE: We agree that our findings do not quantify the relative importance of each outcome – this was not our intention, rather we set out to understand what outcomes are of importance to patients/carers and should be measured. As we have outlined, the next stage of this work is to reach consensus about how outcomes should be defined and measured, with the goal of achieving standardisation across centres.

Here's the crux of the matter. If the authors don't aim for intervention outcomes, Then the study isn't appropriate for the medical field.

With this response, it's clear that the authors aren't evaluating the interventions. So, it wasn't genuinely clear what the study's hypothesis is. If the study doesn't have a hypothesis, what's the point of the research? I believe that with this data, the authors won't be able to justify the standardization of the next study.

VERSION 2 - AUTHOR RESPONSE

Reviewer: 1
Dr. Julie Menzies, Bristol Royal Hospital for Children, United Hospitals Bristol and Weston NHS Foundation Trust
Comments to the Author:
Thank you for the opportunity to review this revised manuscript. The authors have addressed all of my comments / questions and I feel the revisions enhance the paper. I have no further comments / requests. I look forward to seeing this in print.

Thank you for your positive feedback and for confirming that all previous comments have been addressed. We agree that the revisions enhance the paper and are grateful to the reviewer for their engagement with our manuscript.

Reviewer: 2
Dr. Whady Hueb, Universidade Federal de Sao Paulo
Comments to the Author:
To the Authors
The study, as presented, displays an inappropriate format for a medical journal. The title is colloquial, there is no hypothesis, the objective is unclear, the results do not adhere to methodological rigor, and the discussion does not interpret the results. Additionally, there are important limitations, especially selection bias, limited representativeness, and the interpretative qualitative nature of the findings. This restricts the generalizability of the findings. Thus, the results should be considered exploratory and descriptive.

Major problems;

Unresolved issues:

1-TITLE: The title is inappropriate. It is colloquial. It should be changed.
RESPONSE: We have added the study design to the title as requested by the Editor.

Question: I think this response is inappropriate. Unless the editor is also a reviewer.
Usually, the title should have clarity, precision, and scientific relevance.
As a reviewer, I consider the title to be like a newspaper headline.

We respectfully disagree with the reviewer and believe that the title does capture the research question and study design, as stipulated in the author instructions for *BMJ Open*.

2-ABSTRACT: The abstract does not conform to standard abstracts. It should be changed.
RESPONSE: We have followed the abstract structure for BMJ Open but have rewritten the design and setting to include the study design more specifically, as requested by the Editor and added a section on the analysis.

As a reviewer I consider unsatisfactory response. Again. I can't believe the editor agreed to change the abstract format.

We have followed the structure of the abstract for BMJ Open as set out in the author guidelines so we are not clear as to what the reviewer means by the editor agreeing to change the abstract format.

We are happy to change the abstract format if the editor requests it.

3-INTRODUCTION: The introduction should contain a literature review in accordance with the model of this study. It should contain everything that is known about the topic, everything that is not known, its similarities and controversies, and everything that one wants to know.

RESPONSE: Thank you for these comments. We have included literature about congenital heart disease and the measurement of outcomes, highlighting the gap in the literature about what outcomes patients and their parents/carers think are important and should be measured, which is what we describe in this manuscript. We have contextualised the current manuscript in terms of the wider study of which this formed a part, outlined in Figure 1.

As a reviewer, I consider the question unanswered. The authors did not substantially change the introduction.

We agree that we did not substantially change the introduction but we did cover relevant literature about congenital heart disease and the measurement of outcomes, highlighting the gap in the literature about what patients and parents/carers think are important and should be measured. This gap is what we set out to address and is the focus of this manuscript and we do believe that we have provided the reader with what they need to know to understand the context of our work, including detail of the wider study of which this formed a part.

4-OBJECTIVES: The objectives should be clear and assertive.
RESPONSE: Thank you – we appreciate that these may not have been clear so have added a heading for objectives and added the specific objectives addressed in this manuscript.
The objectives are still unsatisfactory. They should be completely redone.

The objectives for this study have been clearly stated and are what we set out to address in the study. We therefore do not believe that 're-doing' the objectives is appropriate.

5-METHODS: The methods should adhere to the rigor of the research. The following is observed:
A- The inclusion of participants occurred through Facebook groups administered by patient organizations, which generates several problems............................
RESPONSE: We recognise all of the points that the reviewer has made and have added further information to the limitations to further address some of these points. Whilst we acknowledge the limitations of the online forum format, we have successfully used this approach before to elicit the views of multiple participants including those who might otherwise not participate in research. As this is a qualitative approach we did not want to 'control' the quality of contributions or achieve equal participation between groups or individuals and we did not want to objectively measure anything. However, we have added the fact that findings are liable to interpretation bias. We have also added further information in the abstract about the approach to analysis. We also acknowledge the limited generalisability to participants in the UK and the UK health system – but would also highlight that our focus was the UK and the UK health system.

Not answered: The authors must understand that the format of a medical publication has strict guidelines and does not allow for shortcuts. This study is not structured according

to the methodological rigor namely: Hypothesis-Objective and Methods. Each of these has its own rules and does not accept changes according to the nature of the study. The fact that the material has already been published, in part, does not give a "free pass" for another publication.

The reviewer's evaluative framework (positivist and quantitative) is clearly different to the methodological framework of our study, which is interpretivist and qualitative, and we respectively suggest that this mismatch has resulted in feedback which cannot be addressed for the submitted work.
The reviewer has identified that there is no hypothesis and at point 7 asks, 'if the study does not have a hypothesis what is the point of research?' Qualitative research is **inherently hypothesis-generating**, not hypothesis-testing and it addresses research questions, not hypotheses. **This is a foundational distinction between paradigms** and is explicit in every major qualitative reporting guideline, including the SRQR which BMJ Open recommends and which we have followed in our reporting.

We have clearly set out our objectives and provided a summary of our methods. We do not subscribe to the view that we have employed 'short cuts' but do accept that as detailed methods have been reported previously we have provided a shorter summary of the methods in the current manuscript (which included the recommended elements on the SRQR reporting guideline).

6- RESULTS
The results reveal an important aspect: there is a discrepancy between the outcomes valued by researchers and those considered relevant by patients.
While traditional research prioritizes: mortality, complications, and reinterventions.
Patients and caregivers highlighted less relevant data: quality of life, psychological impact, uncertainty about the future, access to and organization of care, and adequate information for decision-making.
Another point highlighted is the balance between information and emotional impact.
Many participants highlighted that: insufficient information hinders informed decisions; excessive information can generate anxiety and psychological suffering.
This aspect suggests the need for individualized medical communication strategies.
RESPONSE: Thank you for these comments. Whilst we agree that researcher and patient priorities are likely different, we do not agree that patients and carers highlighted less 'relevant' data – these are important and relevant to them and as such it is important that we measure these outcomes as well as more 'traditional' outcomes. We do agree, however, that individually tailored approaches to communication are needed.

This reviewer disagrees. Patients and caregivers are not medical professionals. They have different focuses. Therefore, the reader of a medical journal is a doctor. They want medical data. Information from caregivers or patients fails to provide technical information.

We strongly disagree with the stance that the reviewer is taking when they state, "*the reader of a medical journal is a doctor. They want medical data. Information from caregivers or patients fails to provide technical information*," and that the study is therefore not appropriate for a medical journal.

This position is incompatible with *BMJ Open*'s stated remit, with the publisher's wider commitment to patient and public involvement, and with the 2016 open letter to the BMJ signed by 76 senior researchers explicitly warning against precisely such dismissal of qualitative and patient-perspective research (Greenhalgh et al., 2016*). It is also incompatible with the now-routine clinical and regulatory emphasis on patient-reported and patient-prioritised outcomes, which is the substantive contribution of our paper.

*Greenhalgh, T., Annandale, E., Ashcroft, R., Barlow, J., Black, N., Bleakley, A., et al. (2016). An open letter to The BMJ editors on qualitative research. BMJ, 352, i563. https://doi.org/10.1136/bmj.i563

7-FINAL CONSIDERATIONS:
Clinical impact and applicability:
Direct applicability in clinical practice is limited because: it does not quantify the relative importance of each outcome; it does not establish measurable metrics; and it does not assess the association between outcomes and prognosis. Therefore, the results should be interpreted as exploratory and hypothesis-generating.
RESPONSE: We agree that our findings do not quantify the relative importance of each outcome – this was not our intention, rather we set out to understand what outcomes are of importance to patients/carers and should be measured. As we have outlined, the next stage of this work is to reach consensus about how outcomes should be defined and measured, with the goal of achieving standardisation across centres.

Here's the crux of the matter. If the authors don't aim for intervention outcomes, Then the study isn't appropriate for the medical field.
With this response, it's clear that the authors aren't evaluating the interventions. So, it wasn't genuinely clear what the study's hypothesis is. If the study doesn't have a hypothesis, what's the point of the research? I believe that with this data, the authors won't be able to justify the standardization of the next study.

The reviewer is correct, we have not evaluated interventions – and it was never our intention to do so. The request of the reviewer for objective, hypothesis driven measurement assumes a postpositivist paradigm which is a mismatch for the interpretivist framework in which our study is grounded. We note that the focused mapping review of how reviewers respond to qualitative studies by Herber and colleagues (2020)[1] identifies exactly such requests as paradigmatic markers of "an underlying quantitative mindset" being applied inappropriately to qualitative work. Clarke and colleagues' (2025) study[2] of 163 qualitative researchers in Qualitative Psychology documents the same pattern at scale and characterises it as reviewers unfamiliar with qualitative research being "really confidently wrong."

1. Herber, O. R., Bradbury-Jones, C., Böling, S., Combes, S., Hirt, J., Koop, Y., Nyhagen, R., Veldhuizen, J. D., & Taylor, J. (2020). What feedback do reviewers give when reviewing qualitative manuscripts? A focused mapping review and synthesis. BMC Medical Research Methodology, 20(1), 122. https://doi.org/10.1186/s12874-020-01005-y (Open access)

2. Clarke, V., Braun, V., Adams, J., Callaghan, J. E. M., LaMarre, A., & Semlyen, J. (2025). "Being really confidently wrong": Qualitative researchers' experiences of methodologically incongruent peer review feedback. Qualitative Psychology, 12(1), 7–24. https://doi.org/10.1037/qup0000322 (Open access)

Throughout the earlier review process we engaged constructively and made methodological clarifications, including additional information in the abstract about the analytic approach, expanded methodological reporting, and acknowledgement of interpretation bias as a limitation. We have added further methodological detail to the text in this round of reviews but we are **not** willing to redesign the study, generate a hypothesis post hoc, add quantification incompatible with the analytic approach, or remove the patient and carer voices that are the study's substantive contribution — because doing so would compromise the methodological integrity of the work and, in our view, would not result in a better or honest paper.

We are very grateful for your time and the care you have taken with this manuscript. We remain committed to publishing this work in BMJ Open and hope that it will now be considered acceptable for publication.
